# Serum-free freezing media support high cell quality and excellent ELISPOT assay performance across a wide variety of different assay protocols

**DOI:** 10.1007/s00262-012-1359-5

**Published:** 2012-11-09

**Authors:** Helene Filbert, Sebastian Attig, Nicole Bidmon, Bernhard Y. Renard, Sylvia Janetzki, Ugur Sahin, Marij J. P. Welters, Christian Ottensmeier, Sjoerd H. van der Burg, Cécile Gouttefangeas, Cedrik M. Britten

**Affiliations:** 1 III. Medical Department, University Medical Center of the Johannes Gutenberg-University, Mainz, Germany; 2 TRON, Translationale Onkologie an der Universitätsmedizin der Johannes Gutenberg-Universität Mainz gGmbH, Verfügungsgebäude 708 (3. OG), Langenbeckstr. 1, 55131 Mainz, Germany; 3 Research Group Bioinformatics (NG 4), Robert Koch-Institute, Berlin, Germany; 4 ZellNet Consulting, Inc., Fort Lee, NJ USA; 5 Department of Clinical Oncology, Leiden University Medical Center, Leiden, The Netherlands; 6 Cancer Sciences Division, Southampton University Hospitals, Southampton, UK; 7 Department of Immunology, Institute for Cell Biology, Eberhard-Karls University, Tübingen, Germany

**Keywords:** ELISPOT, Cryopreservation, Serum-free, Assay harmonization

## Abstract

Robust and sensitive ELISPOT protocols are commonly applied concomitant with the development of new immunotherapeutics. Despite the knowledge that individual serum batches differ in their composition and may change properties over time, serum is still commonly used in immunologic assays. Commercially available serum batches are expensive, limited in quantity and need to be pretested for suitability in immunologic assays, which is a laborious process. The aim of this study was to test whether serum-free freezing media can lead to high cell viability and favorable performance across multiple ELISPOT assay protocols. Thirty-one laboratories from ten countries participated in a proficiency panel organized by the Cancer Immunotherapy Immunoguiding Program to test the influence of different freezing media on cell quality and immunologic function. Each center received peripheral blood mononuclear cells which were frozen in three different media. The participants were asked to quantify antigen-specific CD8+ T-cell responses against model antigens using their locally established IFN-gamma ELISPOT protocols. Self-made and commercially available serum-free freezing media led to higher cell viability and similar cell recovery after thawing and resting compared to freezing media supplemented with human serum. Furthermore, the test performance as determined by (1) background spot production, (2) replicate variation, (3) frequency of detected antigen-specific spots and (4) response detection rate was similar for serum and serum-free conditions. We conclude that defined and accessible serum-free freezing media should be recommended for freezing cells stored for subsequent ELISPOT analysis.

**Electronic supplementary material:**

The online version of this article (doi:10.1007/s00262-012-1359-5) contains supplementary material, which is available to authorized users.

## Introduction

In contrast to classical treatments in oncology that affect tumor cells directly (chemotherapy, radiation, small molecules, monoclonal antibodies targeting tumor-associated antigens), immunotherapies which aim at inducing T-cell immune responses affect tumor cells indirectly. The broad acknowledgment of these conceptual differences for T-cell vaccine led to a dedicated regulatory guidance for therapeutic cancer vaccines, and the acknowledgment to perform concomitant studies of the magnitude, phenotype and function of vaccine-induced immune responses to better understand the anticipated mode of action and to guide the development of new vaccines [1–3]. Indeed, immunologic monitoring has nearly become a “must have” already at early stages of rational vaccine development. Although it is still under debate which immunologic assays should be applied and whether immunologic monitoring should be performed in the peripheral blood or the tumor tissue, it is a fact that hundreds of laboratories worldwide use ELISPOT assays and flow cytometric analysis to monitor vaccine-induced immune responses in peripheral blood mononuclear cells (PBMCs). In addition, an increasing number of reports confirm a correlation between the results of T-cell immune assays and clinical events, which suggests that immunologic monitoring in the peripheral compartment will remain to be important and should be applied complementary to assays in the tumor tissue [4–7].

The Immunoguiding Program of the Cancer Immunotherapy Association (CIMT-CIP) together with the Cancer Research Institute’s Cancer Immunotherapy Consortium (CRI-CIC) initiated a large-scale proficiency testing program for the most commonly used T-cell assays and over the last 8 years established the concept of immune assay harmonization in a field-wide effort including more than 100 laboratories [8, 9]. Past proficiency panels have focused on various aspects of the ELISPOT technology including first harmonization guidelines for assay conduct [10, 11], recommendations for response determination [12], a framework for structured reporting of T-cell assay results [13], as well as systematic studies of the impact of different test media on assay results [14, 15]. Indeed, two independent proficiency panels conducted by CIP and CRI-CIC and a third study from the infectious disease field showed that serum-free media can support excellent assay performance in the ELISPOT assay [16].

**Fig. 1:**
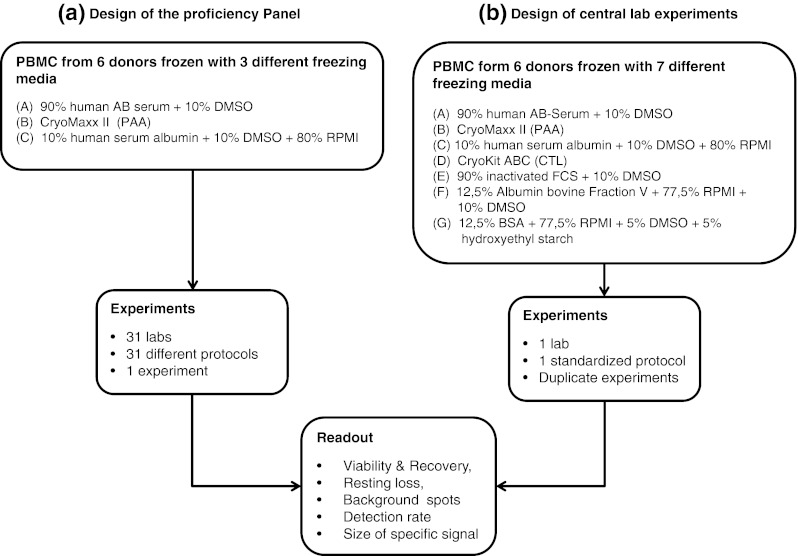
Overview of experiments. The experimental design of the study is depicted as a *flow chart* indicating the starting sample specimens and freezing media applied (two *boxes* in the *top*). Performed experiments were **a** either conducted in a proficiency panel with 31 participating laboratories comparing cells frozen with three different media or **b** in the central laboratory comparing cells frozen with seven different media. The two *boxes* in the center of the *flow chart* indicate the number of investigators that did the experiments, the number of assay protocols that were used and the number of replicates for each experiment. The *box* at the *bottom* indicates the experimental readouts that were made in all experiments and are reported in the “Results” section

In continuation of this systematic and field-wide effort to harmonize ELISPOT assay, CIP in cooperation with CIC organized a proficiency panel to test the impact of serum in the medium used for freezing cells prior to the assay. Good reasons to replace serum in freezing media come from the fact that available batches of human or fetal calf serum (1) consist of non-characterized mixtures of constituents that influence function and phenotype of cells, (2) need to be pretested prior to use, (3) are only available in limited amount which impairs comparability of results generated with cells that were in contact with different serum batches, (4) change their properties during storage and (5) may cause significant delays when frozen cells are shipped across countries due to requirements for import of serum constituents. Consequently, we wanted to compare the viability, cell recovery and functional properties of PBMCs frozen in serum-supplemented or serum-free media. To this end, we conducted an ELISPOT proficiency panel comparing three different freezing media in a group of 31 participating laboratories (Fig. 1a). In addition to the three freezing media that were tested in the proficiency panel, we generated data on an expanded list of seven freezing media in a single-center setting (Fig. 1b).

## Materials and methods

### Organizational setup

The ELISPOT proficiency panel was conducted with a group of 31 centers. Twenty-six participating laboratories were located in 9 European countries (Denmark, France, Germany, Italy, The Netherlands, Spain, Sweden, Switzerland and the United Kingdom). Fourteen of these laboratories were prior participants of CIP proficiency panels, and twelve laboratories participated for the first time. In addition, five laboratories (4× US and 1× Germany) were recruited from the CRI-CIC proficiency panel program that collaborated in this study. Each laboratory received an individual laboratory ID number and was assigned to one of three subgroups of similar size (10, 12 and 10 laboratories, respectively). One participating laboratory analyzed PBMCs from all three subgroups and generated three completed independent data sets. One participant observed an enormous background spot production in all tested donor–antigen combinations, which made the evaluation of the results impossible. This data set was therefore excluded from the final analysis. Consequently, we obtained 32 evaluable data sets from the 31 participating laboratories. The following “Materials and methods” section was prepared compliant to the MIATA guidelines for structured reporting of T-cell experiments [13].

### Sample

PBMCs were isolated from buffy coats obtained from thirteen healthy HLA-A*0201 donors after informed consent at the Transfusion Center, University Medical Center Mainz, Germany. Within 24 h after collection, PBMCs were separated by Ficoll gradient centrifugation and cryopreserved in three different freezing media at 15 million (Mio) cells per vial (A = 90 % heat-inactivated human AB serum (pooled from blood donations from local donors) + 10 % DMSO, B = CryoMaxx II (PAA, Pasching, Austria), C = 10 % human serum albumin (CSL Behring, Marburg, Germany) + 10 % DMSO + 80 % RPMI (Gibco Invitrogen, Darmstadt, Germany) using an automated controlled-rate freezing device (Sy-Lab 14S-B, Neupurkersdorf, Austria). The three media were selected based on results from the survey asking for preferences in participating laboratories.

PBMCs were transferred to the vapor phase of liquid nitrogen and stored until shipment on dry ice to European laboratories (2–20 h transfer time) or shipment in liquid nitrogen shippers for the four US laboratories (32–56 h transfer time). Shipped PMBCs were stored at −80 °C after receipt and thawed after duration of 2–12 weeks at the day of the experiment.

All donor PBMCs were thawed and pretested at least 2 times in IFN-gamma ELISPOT for reactivity against the HLA-A*0201-restricted model epitopes hCMVpp65_495–503_ (NLVPMVATV), FLU M1_58–66_ (GILGFVFTL) and EBV BMLF1_280–288_ (GLCTLVAML). Six donors were selected based on a cell viability of >90 % as determined with a Guava counter in at least 2 independent thawed samples for all three selected freezing medium conditions. Distributed samples in each of the three subgroups were confirmed to have reactivity in four different donor–antigen combinations.

Each participating laboratory received PBMCs from two of the six preselected donors, each frozen in the three different freezing media (A, B and C) and three peptides (CMV, FLU, EBV) for antigenic stimulation. Participants had to thaw all cells using their preferred thawing procedure and determine the number of recovered PBMCs as well as the viability (%) after thawing and resting (a resting phase was recommended but not mandatory for laboratories that have SOPs that do not utilize a resting phase). Eighteen centers performed manual counting using a microscope and Trypan blue exclusion, 11 centers used Guava Counters, and three centers used other methods (CD45/7AAD, Nexcelom Cellometer, Vi-Cell XR). Results obtained for cell viability and recovery in the three tested medium conditions were compared using an unpaired, two-sided *t* test (*p* = 0.05).

For experiments performed at the central laboratory (Mainz), PBMCs from six healthy HLA-A*0201 buffy coats donors (donors 1–6) were collected after informed consent was obtained. The buffy coats were obtained from the Transfusion Center, University of Mainz, Germany. Within 24 h after collection, PBMCs of each donor were separated by Ficoll gradient centrifugation and cryopreserved in 7 different freezing media at 16 × 10^6^ cells per vial: the first three freezing media (A, B and C) correspond to the freezing media tested in the proficiency panel (see above). In addition, the following four freezing media were utilized: (D) CryoKit ABC (CTL, Bonn, Germany), (E) 90 % inactivated FCS + 10 % DMSO, (F) 12.5 % Albumin bovine Fraction V (Serva, Heidelberg, Germany) + 77.5 % RPMI + 10 % DMSO, (G) 12,5 % BSA + 77.5 % RPMI + 5 % DMSO + 5 % hydroxyethyl starch (Fresenius Kabi, Bad Homburg, Germany). PBMCs were frozen using an automated controlled-gradient freezing device (Sy-Lab 14S-B, Neupurkersdorf, Austria) and then transferred into the vapor phase of liquid nitrogen.

### IFN-gamma ELISPOT assay

Participants were asked to quantify antigen-specific T-cell responses against the three peptides (stock solution at 1 μg/μl in 10 % DMSO) that were shipped together with the PBMCs on dry ice to the European laboratories and in liquid nitrogen to the 5 US laboratories. Peptides had to be used at a final concentration of 1 μg/ml. The positive control could be chosen by the participants. In order to facilitate the analysis of data generated, participants received a plate layout that included six replicates of the MOCK control (cells plus medium and no peptide), three peptide antigens added as triplicates and 1 well of positive control for each of the six donor-freezing medium conditions. The laboratories were free to use their own protocol and reagents according to their laboratory SOPs. They had to complete a questionnaire to provide basic information on the ELISPOT operating procedure, such as plates, antibodies, incubation time and staining procedure. For experiments performed at the central laboratory, Multiscreen HA-plates MAHA S45 (Millipore, Darmstadt, Germany) were coated with 50 μl per well of antihuman IFN-γ (7.5 μg/ml, clone Mab 1-D1K, Mabtech) on day 1. The plate was stored overnight at RT. On Day 2, the coating antibody was discarded. The plate was washed 3 times with PBS (Gibco Invitrogen, Darmstadt, Germany) and blocked with X-Vivo (Lonza, Basel, Switzerland) containing 2 % HSA for 1–4 h at 37 °C, 5 % CO_2_. The PBMCs were thawed and the number of recovered PBMCs as well as the viability (%) after thawing and 2-h resting determined. The cells were rested at a concentration of 1 Mio/ml in OpTmizer™ CTS™ T-Cell Expansion SFM (Invitrogen, Darmstadt, Germany) for 2 h at 37 °C, 5 % CO_2_ in 50 ml tubes. The median cell recovery after thawing was 13.3 × 10^6^ with a median viability of 95 %. After resting, the median cell loss was 25.1 %. Cell counts and viability was obtained using a Guava counter EasyCyte 5HT and the ViaCount kit. After resting, the PBMCs were washed and resuspended at 2 × 10^6^ cells/ml in OpTmizer. 150 μl PBMCs per well were added to a final cell number of 300,000 cells per well. 50 μl per well of the peptides hCMV pp65_495–503_ (NLVPMVATV) and FLU M1_58–66_ (GILGFVFTL) were added as triplicates. SEB was added to one well as positive control to a final concentration of 1 μg/ml. In six wells, cells plus medium was added, without peptide (medium control). The plates were incubated at 37 °C, 5 % CO_2_ overnight. On Day 3, the plate was washed and 60 μl per well of the detection antibody Biotin antihuman IFN-γ (1 μg/ml, clone Mab 7-B6-1, Mabtech) was added. After 2-h incubation at 37 °C, 5 % CO_2_, the plate was washed and 100 μl per well of the enzyme avidin-alkaline phosphatase (1:100, Sigma) was added. After 1-h incubation at RT and washing the plate, 100 μl per well of the BCIP/NBT (Sigma) was added according to the manufacture’s instructions. After 3–5 min, the staining reaction was stopped by washing the wells under running water. No internal assay controls were used except for six medium control wells to determine the background spot production.

### Data acquisition

Participants analyzed the plates using their preferred protocol, hardware and software. The results obtained by the ELISPOT reader were controlled by human auditing in 28 of 31 laboratories. Representative ELISPOT filter plates from the proficiency panel phase and the series of experiments performed in Mainz are shown in supplementary figures 1 and 2.

For experiments performed in the central laboratory, the filter plates were analyzed with the CTL ELISPOT reader using the ImmunoSpot 5.0.3 software and a locally established SOP for plate reading. The results obtained by the reader were verified by human auditing. A representative data set is shown in supplementary figure 2.

### Analysis of data

The ELISPOT analysis was performed based on the spot numbers reported by the participants.

For experiments performed at the central laboratory, median background reactivity was 2 spots per 100,000 cells, with a range of 0–33 spots. Antigen-specific spots were determined by subtracting the mean spot number in the six medium control wells from the mean spot number in the experimental triplicates. The response determination in this panel was made using a previously published approach for response determination (*p* value of <0.05 [12]. A Web-based interface for facilitated response determination can be found at http://www.scharp.org/zoe/runDFR/. Raw data of all experiments can be provided upon request.

### Laboratory environment

Participating laboratories operated under different principles, varying from exploratory research to good clinical laboratory practice (GCLP) and good laboratory practice (GLP). Some laboratories used established laboratory protocols, and other laboratories worked with standard operating protocols (SOPs). Most participants reported to be experienced in the ELISPOT technology. Only two participants had no experience.

The central laboratory is working under exploratory research conditions. Work steps for PBMC preparation (cell isolation, freezing, thawing) were performed using laboratory SOPs. The cell staining protocol and filter plate analysis were performed per SOP. The ELISPOT assay protocol was qualified prior to use. To this end, the standardized assay was used in series of experiments with more than 20 donors to define the expected background spot production and intra- and inter-assay variation in the hands of defined operators. All experimental steps from handling of starting material through testing and acquisition of data were conducted by the same experienced operator.

## Results

### Impact of different freezing media on the cell viability and recovery across institutions

**Fig. 2:**
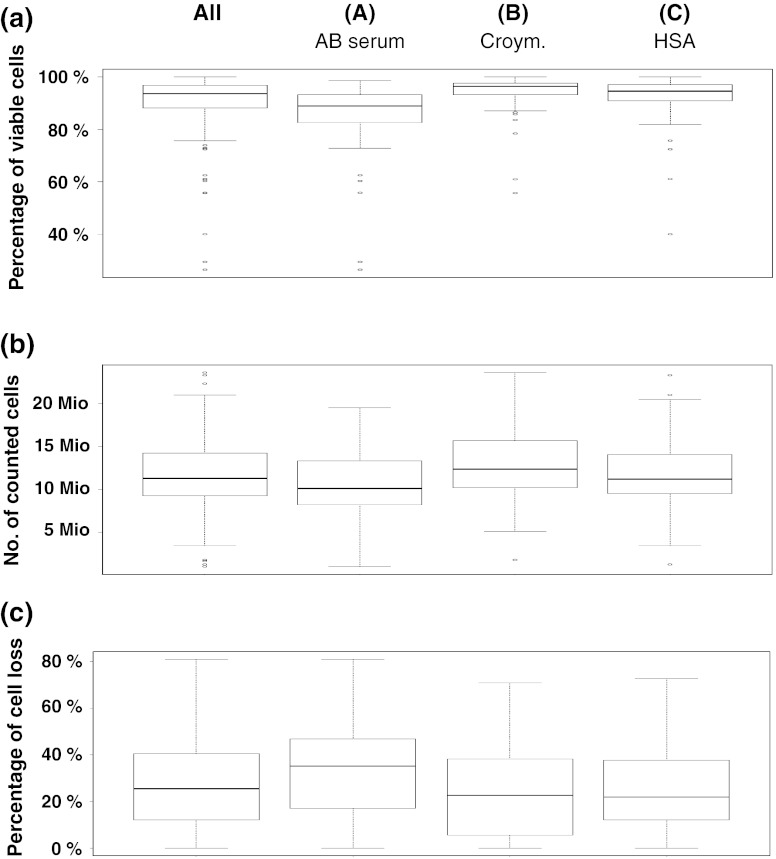
Viability, recovery and resting loss in the proficiency panel. To illustrate the distribution of recovered cells, viability and resting loss for the different freezing conditions *box* plots were used. The *rectangle* shows the interquartile range ranging from the first quartile (the 25th percentile) to the third quartile (the 75th percentile). The *whiskers* point at the minimum and maximum value unless the distance from the minimum value to the first quartile is more than 1.5 times the inter-quartile range (IQR). In that case, the *whisker* extends out to the smallest value within 1.5 times the IQR from the first quartile. The *circles* indicate outliers, which are smaller or larger than the *whiskers*. The *lines* inside the *rectangle* show the median. The *box* plots show the results obtained for all media and stratified by freezing medium conditions *A* (90 % human AB serum + 10 % DMSO), *B* [CryoMaxx II (PAA)] and *C* (10 % human serum albumin + 10 % DMSO + 80 % RPMI). **a** Viability of cells directly after thawing. Statistical testing (unpaired *t* test) was performed (*A* vs. *B*: *p* < 0.0001; *A* vs. *C*: *p* = 0.0015). **b** Recovery of viable cells per vial directly after thawing. Statistical testing (unpaired *t* test) was performed (*A* vs. *B*: *p* = 0.01; *A* vs. *C*: *p* = 0.046). **c** Cell loss during resting. Statistical testing (unpaired *t* test) was performed (*A* vs. *B*: *p* = 0.068; *A* vs. *C*: *p* = 0.0345)

All participating laboratories received three vials of PBMCs from two donors, each frozen in three different media (A: serum, B: serum replacement, commercial, C: serum replacement, self-made). They were asked to thaw the cells and to record recovery and viability immediately after thawing and a second time after resting if applicable (Fig. 2a–c). The viability of cells immediately after thawing across all participants is shown in Fig. 2a. The viability of thawed cell material was high and in 95 % of cases above 70 %. The overall median viability of cells was 93.6 % (A: 88.9 %, B: 96.3 %, 94.5 %). Importantly, the viability of cells frozen with serum (medium A) was significantly lower (unpaired, two-sided *t* test) compared to medium B (*p* < 0.0001) or C (*p* = 0.0015).The majority of laboratories were able to recover a sufficient number of cells to perform all experiments (Fig. 2b).The median recovery of viable PBMCs per vial was 11.3 Mio cells (A: 10.1 Mio, B: 12.4 Mio, C: 11.2 Mio) and was significantly lower for cells frozen in serum-supplemented medium A compared to medium B (*p* = 0.01) or C (*p* = 0.046). Twenty-seven laboratories introduced a resting time of 1–24 h before adding cells to the ELISPOT plate. The median cell loss after resting was 25.5 %, which is in the range of what is typically expected based on the experience of the central laboratory (Fig. 2c). For medium A, the median cell loss was 35.2 %, for medium B 22.6 and 21.9 % for medium C, respectively. Similar results were observed for all six donors. The cell loss after resting for cells frozen with medium A was significantly higher compared to medium B (*p* = 0.0086) or C (*p* = 0.0345). The median viability of cells after resting was 88 % and similar for all three conditions (data not shown). These results demonstrate that selected serum-free freezing media can support a high recovery of viable cells after thawing and resting across multiple different thawing protocols.

### Impact of different freezing media on the immunologic function across different protocols

To determine the impact of the freezing media on the immunologic function after cryopreservation, PBMCs from each donor were tested in an IFN-gamma ELISPOT. Based on the spot counts reported by the participants, the background spot production (medium-only wells), detection rate and replicate variation were determined and analyzed separately for medium A, B or C. Table 1a shows the overall results for the non-specific spot production in the medium control wells for all tested conditions. A similar background in the medium control wells was observed for all three conditions. Apart from one donor, the background spot production in this proficiency panel was low and similar across all laboratories, donors and freezing conditions, with a median frequency ≤1 spot per 100,000 seeded PBMCs.

**Table 1:** Background spot production and detection rates

Freezing medium	Min	25th	50th	75th	Max
(*a*)					
(A) Serum	0.00	0.25	0.75	5.75	125.87
(B) w/o serum (commercial)	0.00	0.00	1.00	2.76	42.37
(C) w/o serum (self-made)	0.00	0.08	0.56	3.36	39.62

	Detection rate	
(*b*)		
(A) Serum	94 of 117	80.3 %
(B) w/o serum (commercial)	108 of 128	84.4 %
(C) w/o serum (self-made)	110 of 125	88.0 %

(a) The background spots found in the medium control wells are depicted as spots per 100,000 seeded PBMCs for all freezing media or stratified by medium conditions A, B and C. The table indicates the minimum and maximum value as well as the 25th, 50th and 75th percentile. (b) The table shows the detection rates of antigen-specific FLU and EBV responses for the three medium conditions

The frequency of CMV-specific spots in five donors (CIP06, 07, 10, 12, 13) was high (>50 spots per 100,000 PBMCs) and hence easy to detect by all participants, or absent (CIP03). Consequently, results generated with the CMV peptide were not considered for the comparison of test performance. Table 1b indicates the accumulated detection rates of antigen-specific FLU and EBV responses across all participants. With medium A, a total of 94 of 117 possible responses were detected (80.3 %), with medium B 108 of 128 (84.4 %) and with medium C 110 of 125 (88.0 %). While the detection rate for medium A was lower than for medium B or C, the difference did not reach statistical significance. Notably, the group of participants was able to detect 84.5 % of all responses which was higher compared to overall detection rates observed in previous panels. Table 2a and b show the number of antigen-specific spots observed in all 6 donors (CIP06, 07, 03, 10, 12 and 13) that were tested in the proficiency panel after stimulation with the FLU (Table 2a) and EBV peptides (Table 2b). Censored means that only those results were considered for this table where the response for the antigen–donor combination of a given laboratory was positive. The table shows that the median and mean number of antigen-specific spots that were reported by participating laboratories across the three different freezing medium conditions were similar.

**Table 2:**
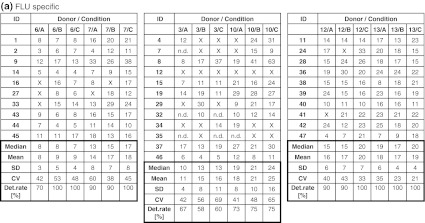

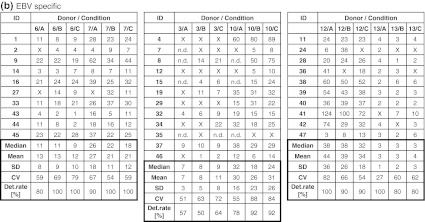
Frequency of (a) FLU-specific and (b) EBV-specific T cells in all subgroups

The table shows the results obtained with each of the six donors (CIP06, 07, 03, 10, 12 and 13) following stimulation with (a) FLU and (b) EBV peptide by all participants. Only replicates that were considered to be above background were considered. All results were normalized to indicate the number of peptide-specific spots per 100,000 seeded PBMCs. The table shows the median, mean, standard deviation and coefficient of variation as well as the detection rates for the twelve different donor–antigen combinations. “X” indicates that the replicate was not considered as being successfully detected. How positive responses were defined in the ELISPOT assay is described in the “Materials and methods” section

*ND* not done

In addition to the background spot production, detection rates and size of the antigen-specific T-cell response, we were interested to determine whether different freezing media may result in differences in the replicate variation (supplementary table 1), which was calculated as variance of the replicate (raw spot counts) divided by (median of the replicate + 1). The replicate variation found in this proficiency panel was similar for the three freezing media conditions. In summary, our results indicate that background spot production, detection rates, size of detected antigen-specific T-cell responses and replicate variation did not vary between the three tested freezing media.

### Impact of different freezing media on the cell viability and recovery within on institution

**Fig. 3:**
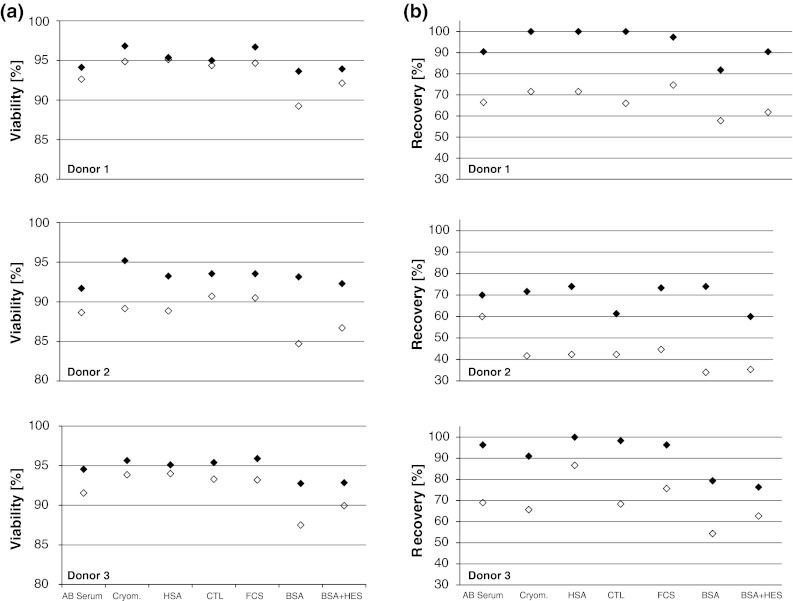
Cell viability and recovery after thawing for seven different freezing media and three donors tested in one center assay. The figure shows results obtained with cells from *donors 1*–*3*. The *filled symbols* show results obtained immediately after thawing. *Open diamonds* show results after resting of cells, prior to testing. **a** Viability of cells (mean result of triplicate at two independent experiments). The quality of cells after thawing and resting was high (median viability 95 %). **b** Recovery of cells (mean result of triplicates from two independent experiments) is indicated as percentage of viable cells that was recovered from each thawed vial relative to the number of cells that were originally filled in each vial

A recent study from Germann et al. [17] showed that cryopreservation media complemented with bovine serum albumin (BSA) and in particular a combination of BSA and hydroxyethyl starch (HES) led to high viability, recovery and functionality of PBMCs in the ELISPOT as compared to PBMCs frozen with 90 % fetal calf serum (FCS). The study also provided evidence that the three freezing media tested by Germann et al. were applicable in the ELISPOT assay with a nearly comparable reactivity. Prior to publication of the Germann study, the organizers of this study focused on human serum albumin (HSA) as a serum replacement. This choice was driven by the fact that lymphocytes were prepared for therapeutic use in adoptive transfer trials. Stimulated by these results, we expanded our tests and tested seven freezing media (described in detail in the “Materials and methods” section), including the two newly proposed serum replacements as well as a FCS-based freezing medium as a comparator. PBMCs from six donors were frozen using the seven different media at 16 × 10^6^ cells per vial. After storage in liquid nitrogen, the cells were thawed and their viability and recovery recorded. Figure 3 depicts the mean of triplicates derived from experiments with donors D1–D3. Results obtained from three additional donors (D4–D6) are shown in supplementary figure 3. The median cell viability after thawing of PBMC from all six donors was 95 % and decreased only slightly after resting (93.5 %), indicating a high quality of utilized cells. The median cell recovery of viable cell from thawed vials was 79 % after thawing which is a high overall recovery rate. Recovery of cells after resting was decreased to 55 % of the total number of the original cell input which indicates that about 30 % of cells that were rested were lost due to the associated handling and washing steps. The results confirm that various serum-free media lead to similar cell viability and recovery as compared to media containing human or calf serum. However, in contrast to the findings from German et al., the media containing BSA or BSA plus HES did lead to the lowest viability and recovery rates after thawing and resting as compared to the other freezing media.

### Impact of different freezing media on the immunological function within one institution

**Fig. 4:**
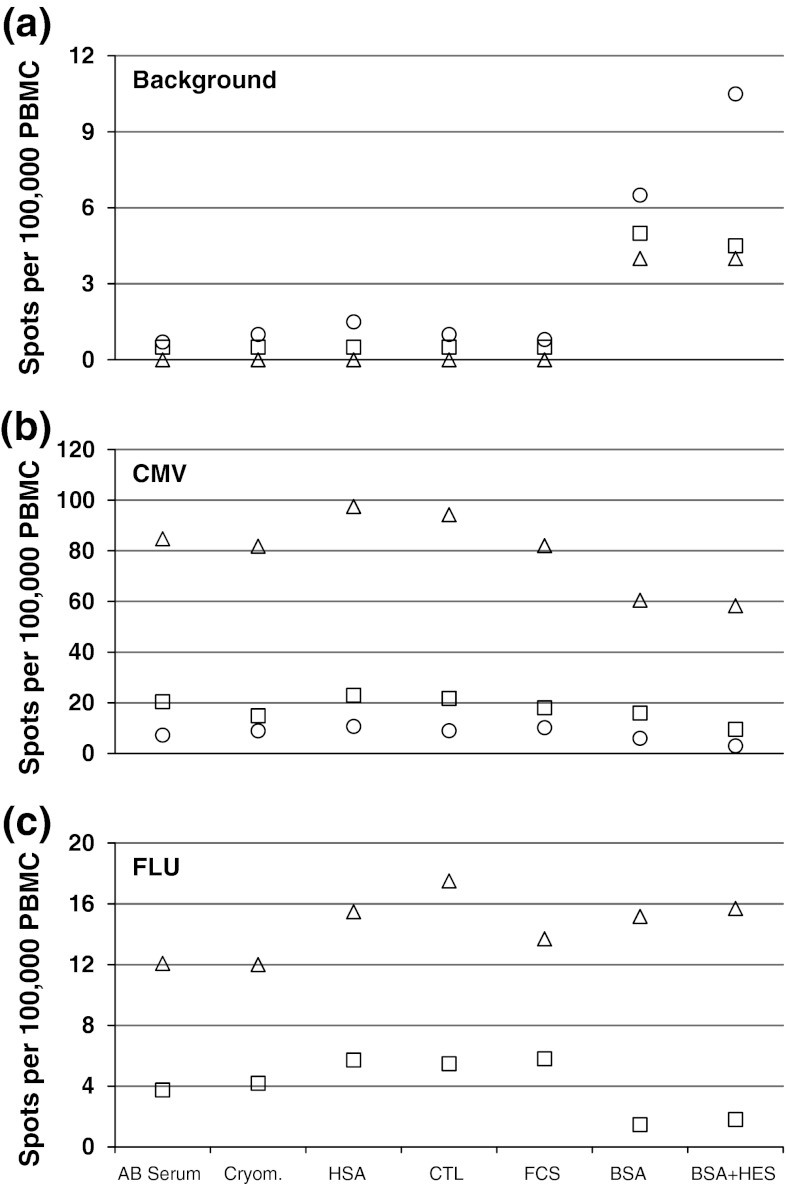
Immunologic function of cells in one center assay. Results are compiled from two independent experiments with cells frozen with seven different freezing media and expressed as mean spot numbers for each of the three donors (*donors 1–3*) tested. Antigen-specific T-cell responses are indicated as spots per 100,000 PBMCs seeded per well. **a** Mean background spot production in the medium control wells. **b** Mean number of antigen-specific spots against the CMV peptide for all three CMV-reactive donors. **c** Mean number of antigen-specific spots against the FLU peptide for the two influenza-reactive donors. *Triangles* D1, *circles* D2, *squares* D3

After thawing and quality control of the PBMC frozen using the seven different freezing media, we tested cells from the six donors in the ELISPOT assay and assessed the background spot production as well as the specific responses against peptides CMV and FLU. Figure 4 and supplementary figure 4 depict the mean spot numbers per donor. The results obtained in this single-center experiment confirm that the serum-free media used in the proficiency panel lead to background reactivity which was comparable to the background spot production induced by freezing media containing human AB serum, but also to other freezing preparations containing or lacking serum (FCS or CTL Cryomedium, respectively). Strikingly, the two media containing BSA or BSA plus HES showed an increased number of spots in the medium control in five of six tested donors (unpaired, two-sided *t* test; *p* < 0.0001). Donor D5 had an unusually high background spot production independent of the utilized freezing medium. Figure 4b and supplementary figure 4b show the specific response against CMV after subtracting the mean background spot numbers from the mean spot numbers in test wells. All six donors were selected as being seropositive and showed a CMV reactivity. All donors except donor D3 also had measurable memory FLU responses (Fig. 4c and supplementary figure 4c). The specific responses against the CMV and FLU peptides for each individual donor were of similar strength for all seven freezing media tested (unpaired, two-sided *t* test). Therefore, we confirmed that cells cryopreserved in serum-free freezing media support detection of similarly sized antigen-specific T-cell responses compared to serum-supplemented media.

## Discussion

The performance of cellular immune assays is influenced by a series of factors including the starting cell material, the assay procedure, the data analysis, the rules applied for response determination and the laboratory environment in which these assays are conducted. Media used in the process, including freezing, thawing, washing and testing of donor PBMCs are critical components. Indeed, multiple studies in the past have shown that serum-free test media for ELISPOT assays that lead to low background, high detection rates and similar magnitude of antigen-specific T-cell responses as compared to media supplemented with pretested serum batches [14, 15]. The extension of this work to a study focusing on freezing media was a consequent next step toward complete removal of serum components throughout the entire process. The data obtained in the current proficiency panel provide evidence that commercially available serum-free freezing medium as well as a self-made serum-free freezing medium supports high cell viability and recovery after thawing and favorable immunologic function in the ELISPOT assay across a multitude of different and highly heterogenous protocols. Obviously, all commonly available freezing media could not be tested in a single proficiency panel, and the selection of three media used in the proficiency panel was made by the participating centers which lead to the fact that a freezing media containing FCS, which is probably the most common supplement used was not included in the proficiency panel. Experiments performed in preparation of the proficiency panel did not indicate differences between cells that were frozen with media supplemented with FCS and human AB serum. Notably, the cell viability and recovery within the panel was excellent, and the background spot production found in this panel phase was lower than expected from previously organized proficiency panels in which cell material that was frozen with media containing FCS was distributed.

A recent single-center study published shortly after the completion of this proficiency panel showed that serum-free media can lead to a high cell quality and immunologic function [17]. Germann et al. used BSA and BSA plus HES as a serum substitute and applied a FCS-based medium as a comparator. As media supplemented with BSA were not included in our proficiency panel, we expanded the list of different freezing media in a series of experiments in a single-center setting and also test FCS-based freezing solution. In contrast to the group of Germann, we found an increased background spot production using cells frozen with a medium supplemented with BSA only or with HES. This may be attributed to use of different donors, antigens tested or protocol properties for thawing, handling and testing the cells in ELISPOT. Independent of the reason for the discrepant findings in these two specific sets of experiments, it remains important to identify freezing media leading to favorable results across a multitude of different assay protocols and regional differences between patient/donor populations. An additional finding of the second part of this study was that all three media used in the proficiency panel led to similar results as compared to cells frozen with a medium that was supplemented with FCS which is broadly used worldwide.

Another single-center study that systematically tested the impact of different freezing media on cell viability and T-cell function compared four different media additives that consisted of (1) fetal bovine serum, (2) autologous plasma and Dextran-40, (3) human AB serum, or (4) human serum albumin [18]. In contrast to what was found in our study, cells frozen in medium supplemented with human AB serum had a decreased viability compared to cells frozen with media containing any of the other three tested additives. This discrepant result may be explained by the fact that different AB serum batches might indeed have different properties. An additional finding of the study from Disis et al. [18] was that cells frozen with a medium supplemented by HSA had a high viability after thawing and supported detection of antigen-specific proliferative responses after stimulation with tetanus toxoid and [3H] thymidine incorporation as readout. This study was the first to suggest that HSA might be a recommendable additive for freezing media for immunologic monitoring assays. Maecker et al. used the optimized freezing medium from Disis et al., which was complemented with HSA (6.25 %) in HLA-peptide multimer staining, cytokine flow cytometry and ELISPOT experiments. Maecker et al. showed that (1) this serum-free medium supported a high sensitivity and specificity in standardized assay protocols and (2) results obtained with frozen cells were similar to the results generated using fresh cell material [19]. An additional study from Bull et al. [20] showed high viability and recovery for freezing media supplemented with HSA and suggested the use of such media for HIV vaccine trials. All these complementary studies support the use of serum-free media that have now been shown by CIP to support favorable cell function across a wide variety of different ELISPOT protocols by our proficiency panel. Additional recommendations for factors that matter when freezing and thawing PBMCs for immunologic assays (e.g., use of warmed medium for initial dilution of cells after thawing) have recently been published as a result of a workshop organized by the Society for Immunotherapy of Cancer and may be considered when optimizing freezing and thawing procedures [21].

Concerning the general use of serum-free freezing media, the authors acknowledge that so far no experimental data exist showing that PBMCs stored in serum-free freezing media over a long storage period do not change properties. In addition, no data exist so far, which indicates how serum-free media impact on the phenotype or function of lymphocytes in other, including flow-based, T-cell assays and on further immune cell populations (e.g., MSDC, NK cells, DCs). In addition, different batches of human serum albumin might contain different impurities which may impact on viability, phenotype and function of cells. Although the variation between different HSA batches will probably be smaller compared to differences of serum batches, a pretesting of new HSA batches may become needed to control assay performance over time [22]. In conclusion, more functional tests following long-term storage of PBMC in serum-free media, similar designed studies for assays studying other immune cell populations and bridging studies prior to changing HSA batches are mandated.

As shown in Table 1b, the evaluable thirty-one laboratories participating in the proficiency panel had reached high detection rates of detecting antigen-specific T-cell responses against the FLU and EBV peptides at low/moderate or even very low frequencies (all <40 spots per 100,000 PBMCs). Such high detection rates for a heterogeneous group of laboratories were not observed in previous panel phases organized by CIP. The observation of such high detection rates was unexpected as most cells provided were probably not frozen in the medium condition that was used to optimize the assay protocols in the individual participating laboratories. Two factors that might have contributed to this detection rate “above average” are that (1) the five CRI-CIC laboratories that participated in this study were known to be top performers in former proficiency panels and (2) 14 laboratories in this panel already participated in previous ELISPOT proficiency panels of CIP. The scans of the filter plates shown in supplementary figures 1a–c show an expected heterogeneity of spot and filter appearance on one hand and an unexpected high consistency of results generated across institutions on the other hand. Again such a high concordance of results was not found in previous proficiency panels. Although the design of the study does not allow to formally prove that the previous participation in harmonization efforts was indeed the reason for the overall high performance in this study group, the authors cannot exclude that the favorable results observed may be due to an increased level of harmonization among participants.

Altogether, it is concluded that the results generated both in the proficiency panel and in the single-center study provide a firm basis for the recommendation to use serum-free media for freezing of PBMCs collected throughout clinical testing. The use of defined media for freezing and testing of PBMCs may lead to a higher reproducibility of results generated over time and across institutions and less delays when importing cell material in multinational trials.

### Electronic supplementary material

Below is the link to the electronic supplementary material.

Supplementary material 1 (PDF 1,185 kb)

## Appendix

Participants of the ELISPOT proficiency panel of the CIP:

1. M. Aigner, S. Standar, A. Mackensen, Department of Haematology and Oncology, University Hospital of Erlangen, Germany
2. S. Heidu, C. Gouttefangeas, Institute for Cell Biology, Department of Immunology, Eberhard-Karls University, Tübingen, Germany
3. M. Subklewe, F. Lichtenegger, Department of Internal Medicine III, University Medical Center, Munich, Germany
4. F. Zhao, A. Paschen, Dermatology, University Medical Center Essen, Essen, Germany
5. D. Maurer, S. Walter, Immatics Biotechnologies GmbH, Tübingen, Germany
6. B. Stadlbauer, H. Pohla, Laboratory of Tumor Immunology, Ludwig-Maximilians University, Munich, Germany
7. D. Riemann, C. Giersberg, B. Seliger, Institute of Medical Immunology, Martin Luther University, Halle, Germany
8. B. Scheel, S. Eppler, CureVac GmbH, Tübingen, Germany
9. H. Filbert°, S. Attig°, C. Britten*, °III. Medical Department, University Medical Center of the Johannes Gutenberg-University, Mainz, Germany, *TRON—Translational Oncology at the University Medical Center Mainz, Mainz, Germany
10. S. Flindt, T. Hinz, Paul-Ehrlich-Institute, Langen, Germany
11. S. Gross, W. Leisgang, E. Kaempgen, Department of Dermatology, University Hospital of Erlangen, Germany
12. N. Grebe, E. Schmitt, Department of Immunology, University Medical Center of the Johannes Gutenberg-University, Mainz, Germany
13. C. Falk°, L. Umansky*, T. Lechl*, *German Cancer Research Center DKFZ, Immune Monitoring Unit, Heidelberg, Germany—Institute for Transplant Immunology, IFB-Tx, Hannover Medical School, MHH, Hannover, Germany
14. G. Moncunill, L. Puyol and C. Dobaño, Barcelona Center for International Health Research (CRESIB), Barcelona, Spain
15. M. Jonassen, M.H. Andersen, Center for Cancer Immune Therapy, Copenhagen University Hospital, Herlev, Denmark
16. M. J.P. Welters. S. H. van der Burg, Department of Clinical Oncology, Leiden University Medical Center, Leiden, The Netherlands
17. R. Maier, Institute of Immunobiology, Kantonal Hospital, St. Gallen, Switzerland
18. G. Di Lullo, M. P. Protti, Laboratory of Tumor Immunology, San Raffaele Scientific Institute, Milano, Italy
19. W. Shingler, G. Morgan, Oxford BioMedica, Oxford, UK
20. B. Näsman-Glaser, I. Poschke, R. Kiessling, Karolinska University Hospital, Stockholm, Sweden
21. S. Man, C. Nunes, Institute of Infection and Immunity, School of Medicine, Cardiff University, UK
22. A. Harenberg, S. Gimenez-Fourage, F. Jantet-Blaudez, Sanofi Pasteur, Department of Non-Clinical Product Performance, Marcy l’Etoile, France
23. X. Preville, R. Rooke, Transgène S.A, Illkirch Graffenstaden, France
24. S. Paulie, I. Areström, Mabtech, Stockholm, Sweden
25. K. Tier, L. Chudley, C. Ottensmeier, Experimental Cancer Medicine Center, Faculty of Medicine, University of Southampton, Southampton, UK
26. C. Bain, N. Anfossi, PLATINE PHARMA SERVICES, Centre d’infectiologie, Lyon, France
27. S.G. Smith, H.M. Dockrell, London School of Hygiene and Tropical Medicine, London, UK
28. D. Morelli, B. Yu, Moffitt Cancer Center, Tampa, USA
29. F. A. Legrand, R. Owen, BN ImmunoTherapeutics, Mountain View, USA
30. A. Valencia, B. Nails, Department of Vaccine and Development, Millitary HIV Research Program (MHRP), Henry Jackson Foundation (HJF), Rockville, USA
31. G. Ferrari, M. Berrong, K. Long, Duke University, Durham, USA. This work was supported by the Collaboration for AIDS Vaccine Discovery (CAVD) award #38650.
