# Supplementary material for: Serum-free freezing media support high cell quality and excellent ELISPOT assay performance across a wide variety of different assay protocols
Source: Cancer Immunol Immunother. 2012 Nov 9;62(4):615–27. doi: 10.1007/s00262-012-1359-5 (PMC3624011; doi:10.1007/s00262-012-1359-5)
Supplement: Supplementary file 1 — Supplementary material 1 (PDF 1,185 kb) [file 262_2012_1359_MOESM1_ESM.pdf]

Supplementary figure 1a: Representative scans of the filter plates submitted by the participants. Group 1: Donor CIP06 + CIP07, Triplicates for MOCK, CMV, FLU, Condition C

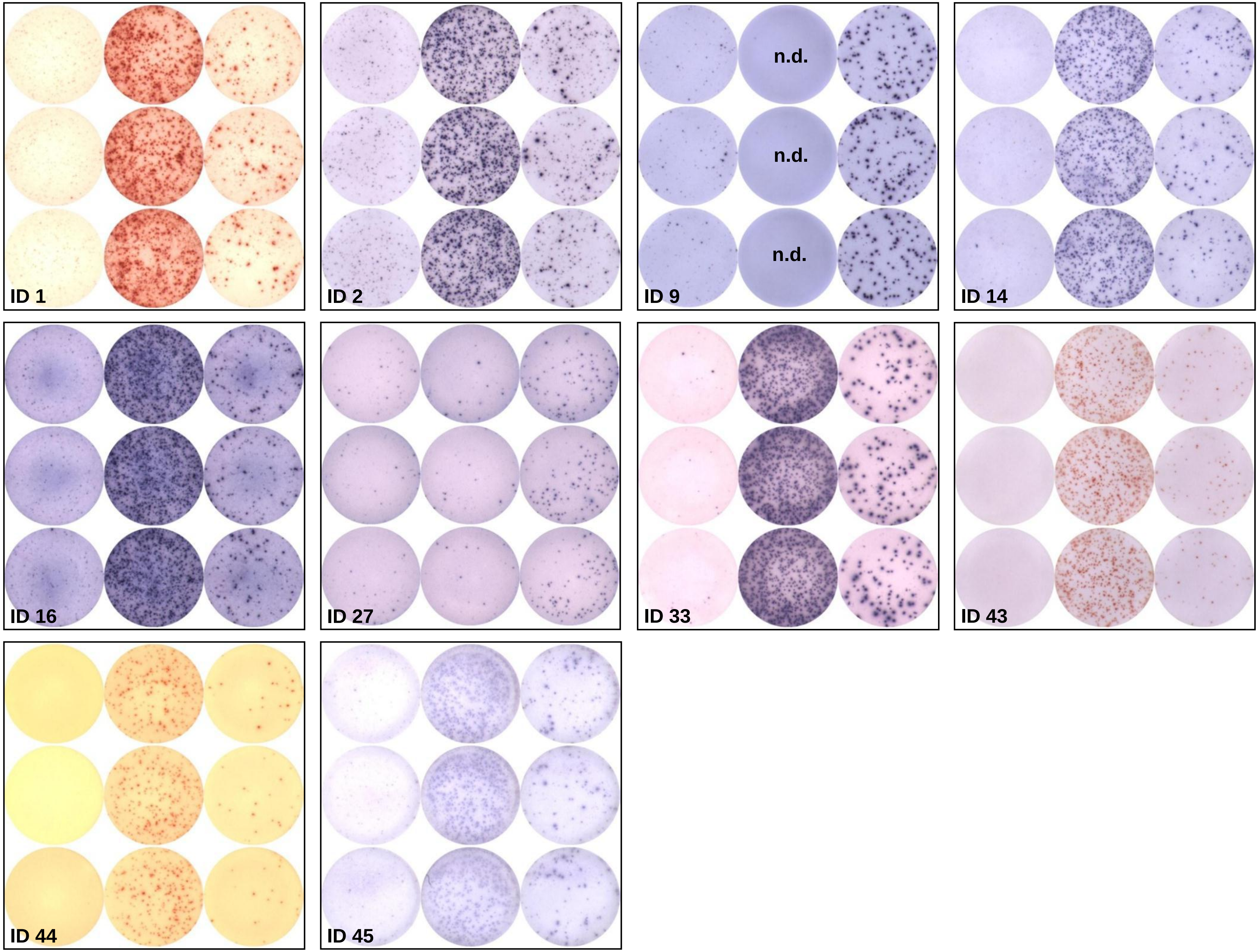

Supplementary figure 1b: Representative scans of the filter plates submitted by the participants. Group 2: Donor CIP03 + CIP10, Triplicates for MOCK, CMV, FLU, Condition C

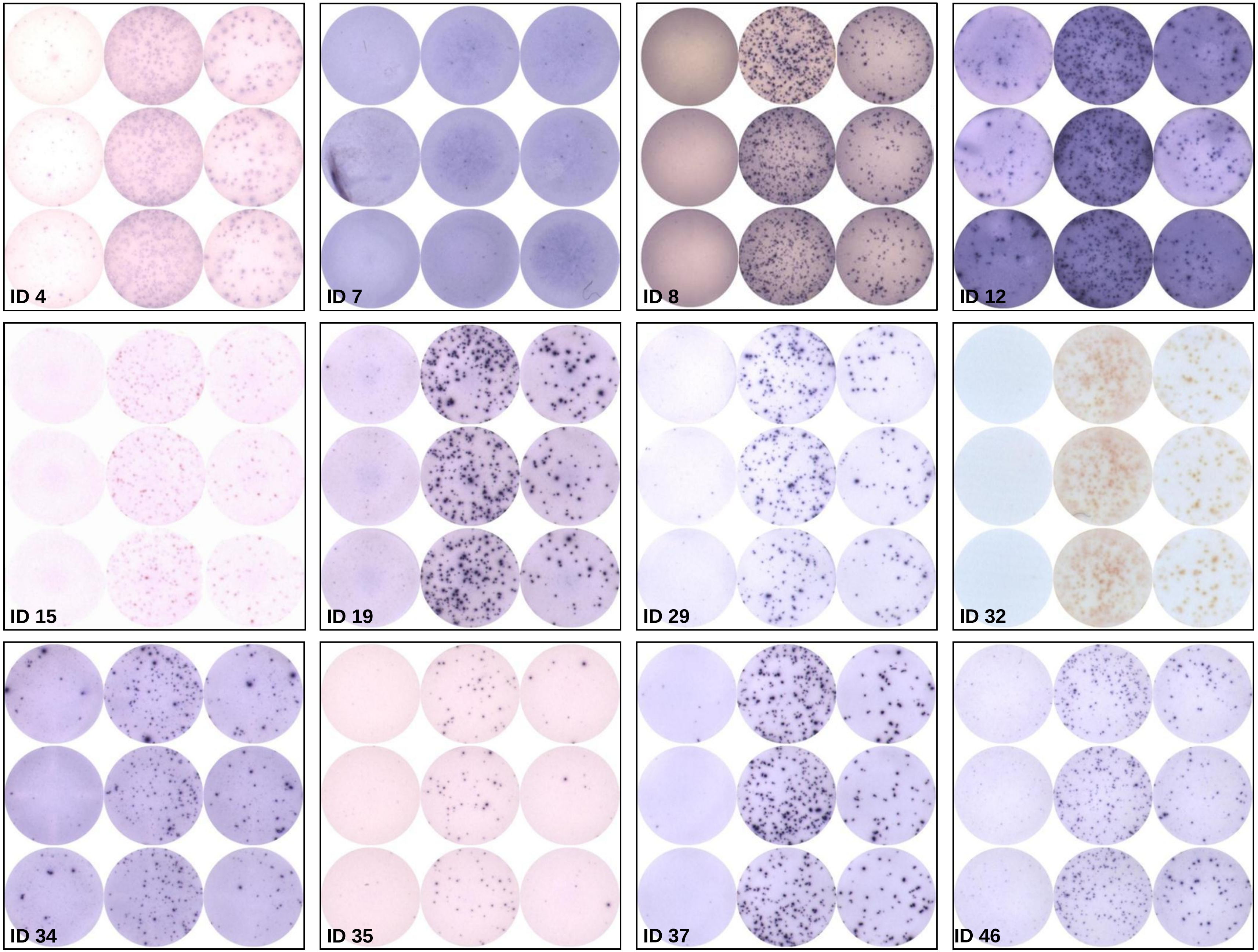

Supplementary figure 1c: Representative scans of the filter plates submitted by the participants. Group 3: Donor CIP12 + CIP13, Triplicates for MOCK, CMV, FLU, Condition C

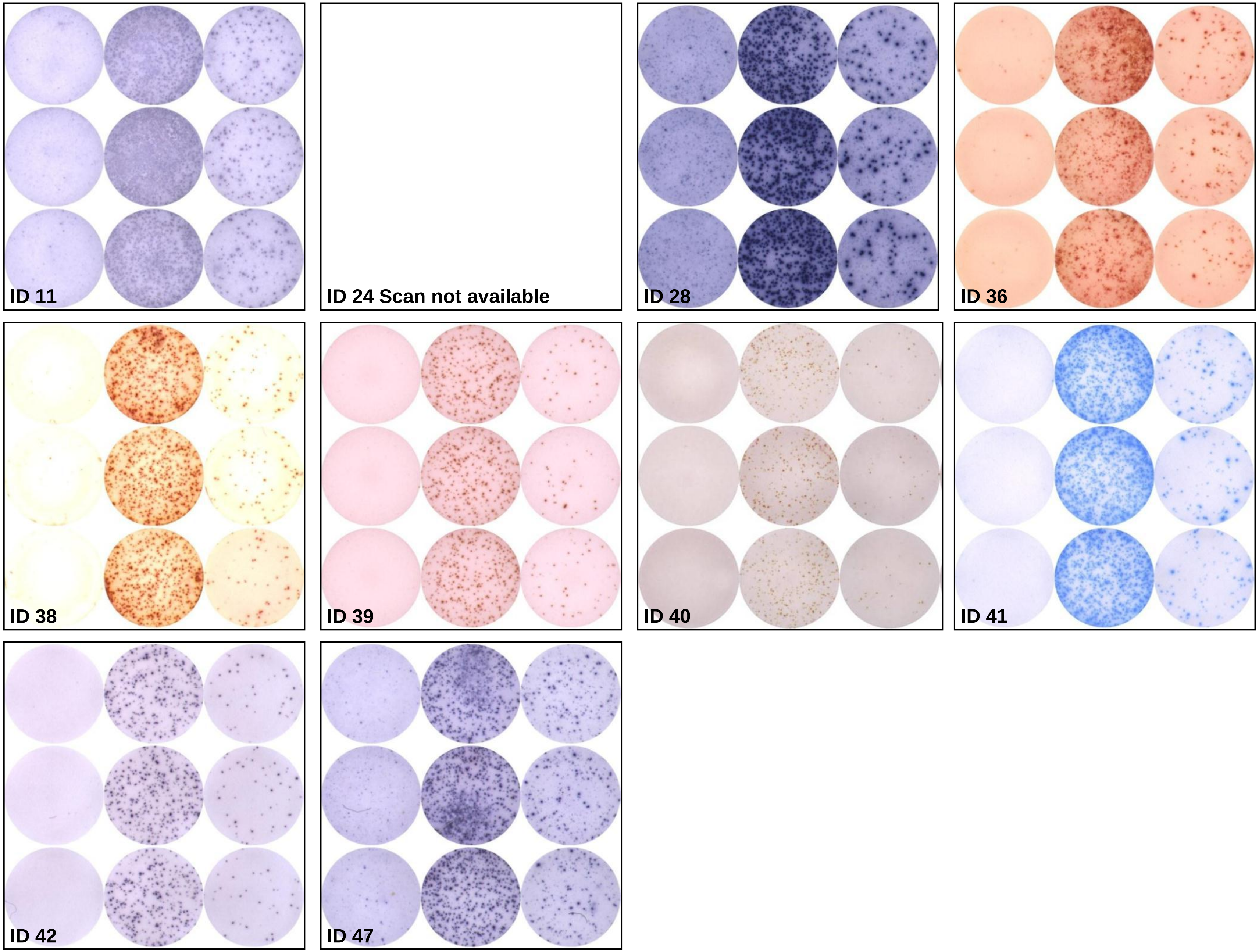

Supplementary figure 2: Representative scans of the filter plates generated in the organizer's lab.

Results from experiments of donor 1. Each scan shows 3 wells after addition of CMV peptide, 3 wells after addition of the FLU peptide, and 6 wells (right column) without addition of any peptide (Medium Control). The experiments were performed in seven replicates with the only difference being the freezing medium. Seven freezing media were used: (A) 90% inactivated AB-Serum + 10% DMSO, (B) CryoMaxx (PAA), (C) 10 % human serum albumin (HSA) + 10 % DMSO + 80 % RPMI, (D) CryoKit ABC (CTL), (E) 90% inactivated FCS + 10% DMSO, (F) 12,5% bovine serum albumin Fraction V (BSA) + 77,5% RPMI + 10% DMSO, (G) 12,5% BSA + 77,5% RPMI + 5% DMSO + 5% hydroxyethyl starch (HES).

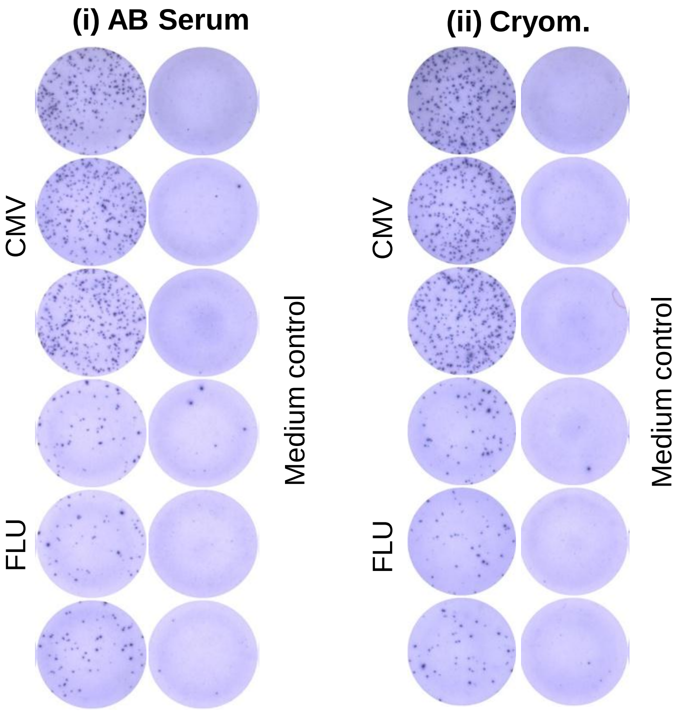

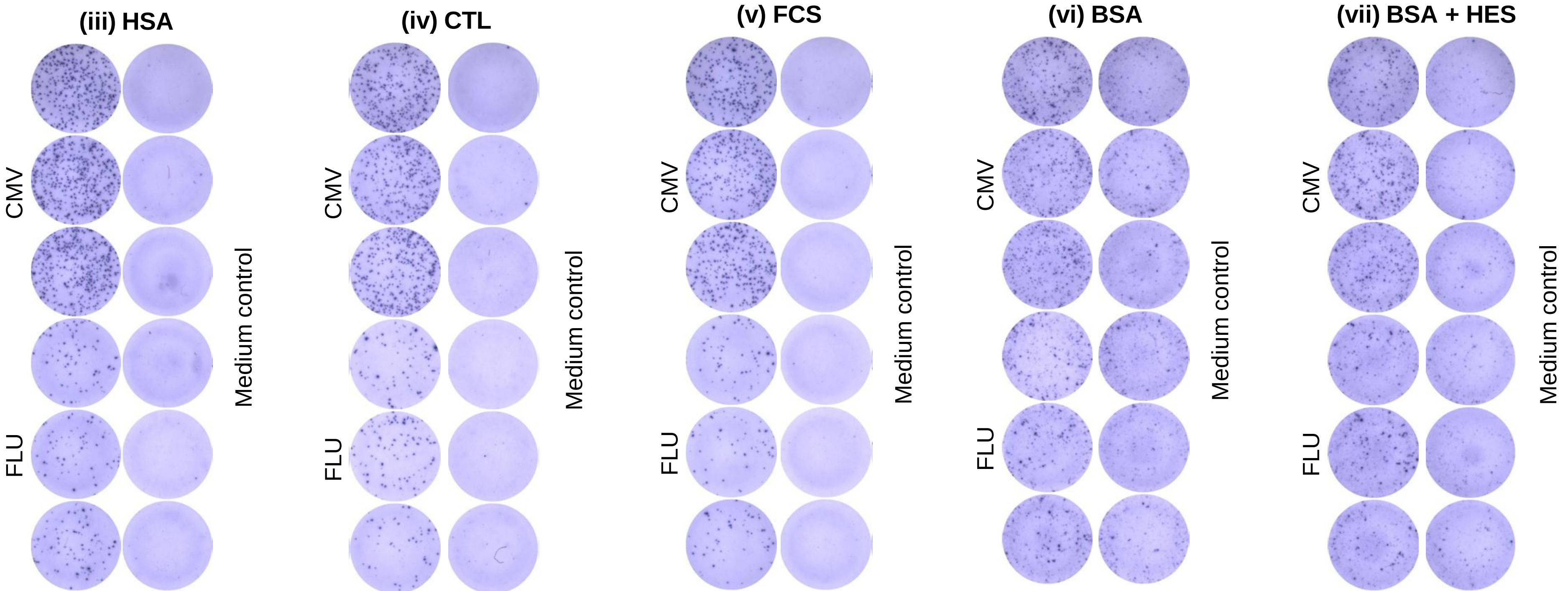

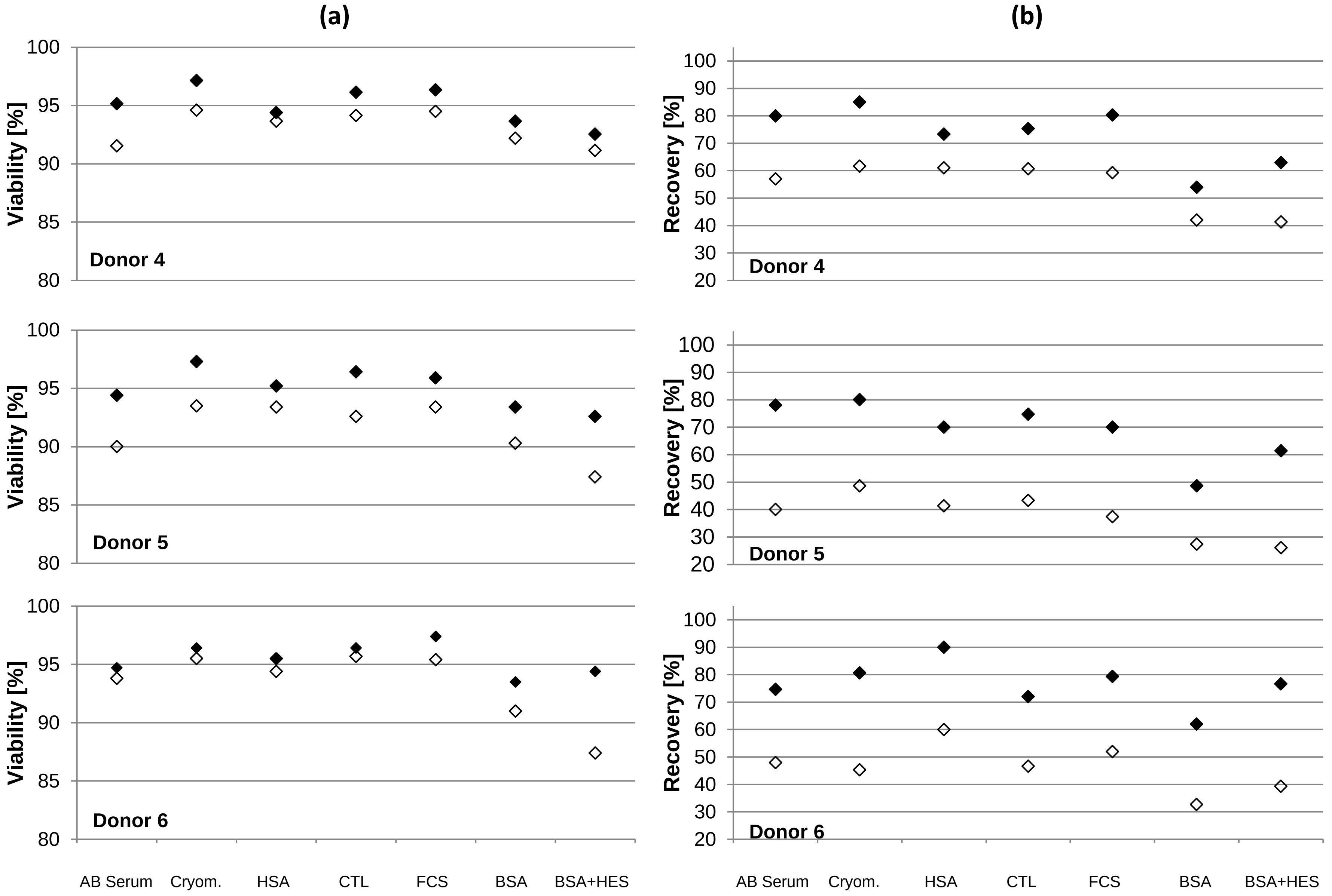

Supplementary figure 3: Cell viability and recovery after thawing for seven different freezing media. Results from three donors (Donors 4-6) are shown. The filled symbols show results obtained immediately after thawing. Open symbols indicate results after resting of cells, prior to testing. **(a)** Viability of cells (mean result of triplicate). The quality of cells after thawing and resting was high (median viability ~95 %). **(b)** Recovery of viable cells (mean result of triplicates) is indicated as percentage of recovered cells relative to the total number of cells that were originally filled.

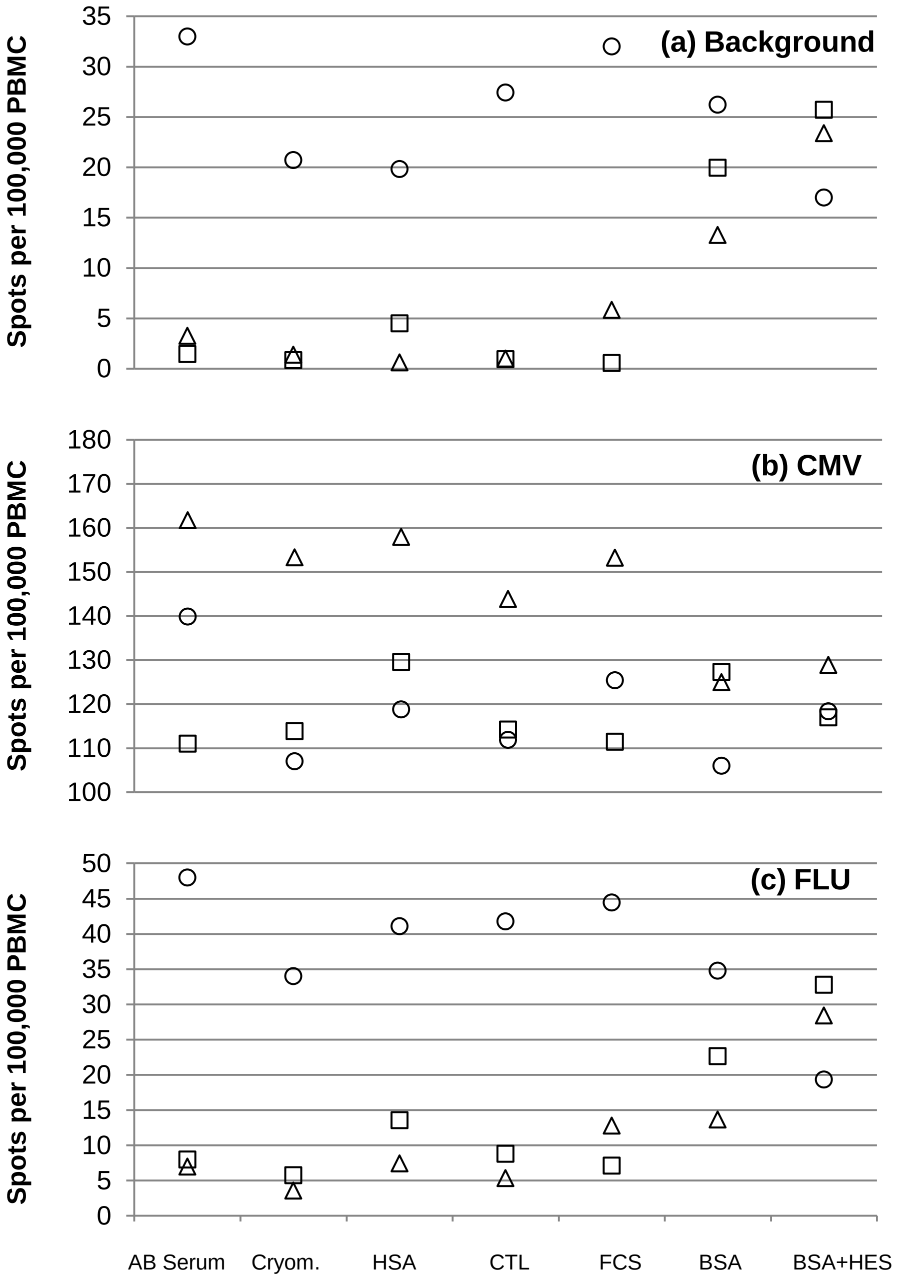

Supplementary figure 4: Immunological function of cells in one center assay. Results from experiments with cells frozen with seven different freezing media are shown and expressed as mean spot numbers for each of the three donors (Donors 4-6) tested**.** Antigen-specific T-cell responses are indicated as spots per 100.000 PBMCs seeded per well. **(a)** Mean background spot production in the medium control wells. **(b)** Mean number of antigen-specific spots against the CMV peptide for all three CMV-reactive donors. **(c)** Mean number of antigen-specific spots against the FLU peptide. Triangles = D4, circles = D5, squares = D6. Donor 5 showed an unusually high background spot production independent of the utilized freezing medium.

Freezing medium	Min	25th	50th	75th	95th
**A) Serum**	0.00	0.12	0.34	0.77	3.82
**B) w/o Serum (commercial)**	0.00	0.09	0.30	0.92	4.59
**C) w/o Serum (self-made)**	0.00	0.11	0.31	0.90	3.90

Supplementary table 1: Replicate variation. The table displays the replicate variation depicted as variance of the replicate (raw spot counts) divided by (median of the replicate + 1).The table shows the minimum value as well as the 25th, 50th, 75th and 95th percentile. The replicate variation was similar for the three freezing media
